# Pasteurella Bacteremia and Otitis Media With Effusion in a Cancer Patient Without Neutropenia: Lessons for Early Diagnosis Through History-Taking and Blood Cultures

**DOI:** 10.7759/cureus.81460

**Published:** 2025-03-30

**Authors:** Momoka Kitani, Kenta Iijima

**Affiliations:** 1 Department of Infectious Diseases, Hyogo Prefectural Amagasaki General Medical Center, Amagasaki, JPN

**Keywords:** bacteremia, breast cancer, non-neutropenic fever, otitis media with effusion, pasteurella multocida

## Abstract

*Pasteurella multocida* often causes zoonotic infections, ranging from mild, localized cellulitis to severe sepsis, and can be life-threatening in immunocompromised patients. However, diagnosis can be challenging due to its varied and nonspecific symptoms. We report a case of *P. multocida* bacteremia and otitis media with effusion in a 49-year-old female breast cancer patient undergoing chemotherapy without neutropenia. The patient presented with a sore throat, which was followed by headache, ear fullness, and fever. She was initially diagnosed with a viral infection and sent home. Two days later, blood cultures revealed Gram-negative coccobacilli, which were later identified as *P. multocida*. The patient reported close contact with dogs but no bites or scratches. Further examination revealed otitis media with effusion. Treatment with ceftriaxone was initiated and subsequently switched from ceftriaxone to ampicillin, then to oral amoxicillin. The patient’s symptoms improved, and she was discharged without sequelae after 10 days of antibiotic therapy. Through a literature review, we identified a high rate of central nervous system complications associated with *P. multocida* acute otitis media, underscoring the necessity for early diagnosis. This case highlights the importance of thorough history-taking, including inquiry about animal exposure, and prompt blood culture acquisition in febrile cancer patients without neutropenia to ensure timely and effective management of potentially life-threatening infections.

## Introduction

*Pasteurella multocida*, a Gram-negative coccobacilli, is a zoonotic pathogen that is commonly transmitted through animal bites, scratches, minimal contact, or licking [1,2]. *P. multocida* commonly causes mild and localized infections, such as cellulitis and sinusitis; however, it can lead to severe systemic diseases, including bacteremia, sepsis, meningitis, and endocarditis [3-6]. Patients with cancer or immunodeficiency are particularly susceptible to severe complications, with mortality rates as high as 20-30% [7]. When *P. multocida *infections offer diverse and nonspecific clinical presentations that mimic more common viral infections, they may further complicate the diagnosis.

There are well-established diagnostic and treatment protocols for febrile neutropenia (FN) in cancer patients, allowing timely management [8]. However, although the majority of cancer patients do not have neutropenia, standardized guidelines for non-neutropenic patients remain insufficient [9,10]. This lack of standardization can lead to delays in recognizing uncommon but potentially life-threatening infections, such as *P. multocida*.

Here, we present a unique case of *P. multocida* bacteremia in a patient with breast cancer without neutropenia who was receiving chemotherapy. The patient presented with fever, symptoms resembling upper respiratory tract infection, and mild otitis media with effusion. This case highlights the importance of incorporating detailed animal contact history and blood cultures into the diagnostic workup for febrile cancer patients. We also conducted a literature review focusing on otitis media as a rare complication of *P. multocida* infection and discussed the diagnostic challenges in non-FN cancer patients with nonspecific symptoms.

## Case presentation

A 49-year-old woman presented to the emergency department with fever, mild headache, joint pain, left cervical pain, and right ear fullness that started that morning. The patient reported a sore throat that had persisted for two days before fever onset. The patient had a history of breast cancer and 9 mm and 8 mm human epidermal growth factor receptor 2-positive and hormone-negative tumors in the upper-outer quadrant of the left breast, with lymph node swelling from the left armpit to the clavicle superior fossa. The tumors were treated with chemotherapy initiated 17 months prior and continued to be treated with partial mastectomy 10 months before presentation. Trastuzumab and a single course of radiation therapy were administered eight months prior. The most recent chemotherapy course was five days before fever onset. The chemotherapy regimen consisted of pertuzumab, trastuzumab, dexamethasone, docetaxel, epirubicin, and cyclophosphamide. The patient had no other significant medical history or regular medications.

**Figure 1:**
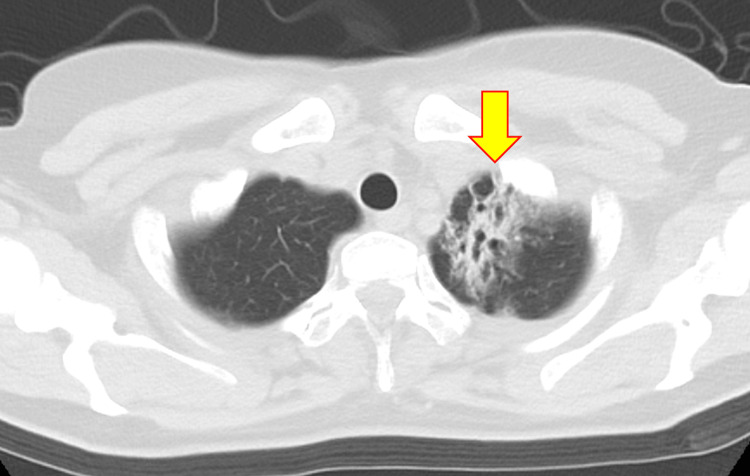
Chest CT scan showing postradiation changes in the left lung apex and upper lobe (yellow arrow).

On physical examination, the patient’s vital signs were as follows: blood pressure, 143/78 mmHg; heart rate, 114 beats per minute; respiratory rate, 25 breaths per minute; and body temperature, 39.3°C. The patient was alert and oriented. Examination revealed mild pharyngeal erythema, with no other obvious source of fever identified. Initial laboratory investigations (Table 1) revealed the following: leukocytes, 10.9 × 10⁹/L (neutrophils 85%, eosinophils 1.7%); hemoglobin, 13.3 g/dL; platelets, 117 × 10⁹/L; aspartate aminotransferase (AST), 53 U/L; alanine aminotransferase (ALT), 32 U/L; alkaline phosphatase (ALP), 156 U/L; lactate dehydrogenase (LDH), 359 U/L; gamma-glutamyl transferase (γ-GTP), 104 U/L; creatinine, 0.56 mg/dL; and C-reactive protein, 1.76 mg/dL. Multiplex polymerase chain reaction (PCR), the BioFire® FilmArray Respiratory Panel (RP 2.1) (bioMérieux, France), revealed negative results for common respiratory viruses, including COVID-19, respiratory syncytial virus, and parainfluenza virus. A cervical and chest CT scan revealed postradiation changes in the left lung apex and upper lobe (Figure 1). Based on these findings, an initial diagnosis of viral infection without febrile neutropenia was made. Two sets of blood cultures were obtained to rule out bacteremia, and 500 mL of lactated Ringer’s solution was administered, resulting in a reduction of the heart rate to below 90 beats per minute. The source of the fever remained unclear based on the examinations performed in the emergency department, and the clinical findings were consistent with an acute upper respiratory tract infection. Although the initial Systemic Inflammatory Response Syndrome criteria suggested a possible risk of sepsis, the readily available medical care in Japan allowed for safe outpatient observation with prompt recall if necessary. Consequently, the patient was discharged home.

**Table 1:** Laboratory investigations at the initial visit and at admission with normal reference ranges. NA: not available

Parameters (units)	Initial visit	At admission	Normal range
White blood cell count (×10^9^/L)	10.9	15.4	3.3–8.6
Red blood cell count (×10^12^/L)	4.66	4.80	3.86–4.92
Hemoglobin (g/dL)	13.3	13.6	11.6–14.8
Hematocrit (%)	40.0	40.4	35.1–44.4
Mean corpuscular volume (fL)	85.8	84.2	83.6–98.2
Platelet (×10^9^/L)	117	133	158–348
Neutrophil (%)	85.0	82.9	43.0–65.0
Eosinophil (%)	1.7	0.0	2.0–5.0
Lymphocytes (%)	8.1	7.9	20.0–50.0
Total bilirubin (mg/dL)	0.5	0.7	0.4–1.5
Aspartate aminotransferase (U/L)	53	41	13–30
Alanine aminotransferase (U/L)	32	28	7–23
Alkaline phosphatase (U/L)	156	157	38–113
Lactate dehydrogenase (U/L)	359	378	124–222
Gamma-glutamyl transferase (U/L)	104	106	9–32
Amylase (U/L)	93	61	44–132
Creatine kinase (U/L)	134	145	41–153
Blood urea nitrogen (mg/dL)	9.3	12.4	8–20
Creatinine (mg/dL)	0.56	0.73	41–153
Glucose (mg/dL)	110	117	73–109
Sodium (mmol/L)	136	136	138–145
Potassium (mmol/L)	3.4	3.5	3.6–4.8
Chloride (mmol/L)	100	96	101–108
C-reactive protein (mg/dL)	1.76	17.5	0.00–0.14
pH (venous gas)	7.435	NA	7.33–7.44
Anion gap (venous gas) (mmol/L)	8.4	NA	5.0–13.0
Lactate (mmol)	1.3	NA	1.0–1.5

**Figure 2:**
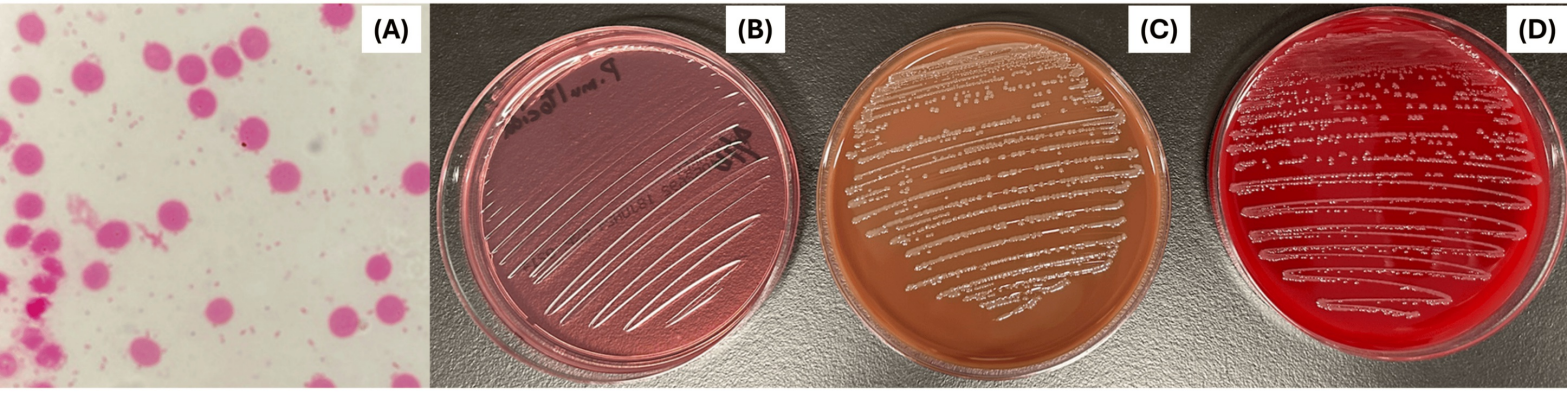
Blood culture and agar results. (A) Gram stain showing Gram-negative coccobacilli in the blood culture under a light microscope at 1,000× magnification. (B) No colony growth on MacConkey agar. (C) Colony growth on chocolate agar (D) and blood agar.

Two days after fever onset, Gram-negative coccobacilli were identified in both aerobic and anaerobic blood culture bottles (Figure 2A). Colony growth was observed on chocolate agar and blood agar but not on MacConkey agar (Figures 2B-2D). Multiplex PCR, the BioFire® FilmArray Blood Culture Identification 2 Panel (BCID2) (bioMérieux, France), revealed negative results for common pleomorphic bacilli, including *Acinetobacter baumannii *and *Haemophilus influenzae*. Upon further inquiry, the patient reported close contact with many dogs but denied receiving any bites or licks. In light of the negative multiplex PCR results for common pleomorphic bacilli, the absence of colony growth on MacConkey agar, and the patient’s history of close contact with dogs, *Pasteurella *species were strongly suspected as the causative agent of bacteremia.

Following the blood culture results, we recalled the patient, and she returned for reassessment. Upon reassessment, the following inflammatory markers had increased significantly (Table 1): leukocytes at 15.4 × 10⁹/L and C-reactive protein at 17.57 mg/dL. Liver function tests revealed slight elevation: AST, 41 U/L; ALT, 28 U/L; ALP, 157 U/L; LDH, 378 U/L; and γ-GTP, 106 U/L. The fullness of the right ear persisted after the patient’s pharyngeal pain subsided. The patient denied earache but reported a subtle headache without nuchal rigidity or altered consciousness. Otorhinolaryngological examination revealed findings consistent with otitis media with effusion, including a mildly erythematous tympanic membrane and minimal effusion behind the membrane without Eustachian tube obstruction.

Based on these findings, we diagnosed the patient with bacteremia with otitis media with effusion. We initiated ceftriaxone 2 g/day. On the fifth day after fever onset, matrix-assisted laser desorption ionization-time of flight mass spectrometry, the MALDI Biotyper® (Bruker, Germany), identified the bacteria as *Pasteurella multocida*. Based on the susceptibility test results (Table 2), ceftriaxone was switched to 2 g ampicillin three times daily. The increased inflammatory responses and symptoms improved progressively following antibiotic therapy. A multidisciplinary discussion with the oncologist, infectious disease physician, and otolaryngologist led to the treatment plan. The infectious disease physician recommended 7-14 days of intravenous antibiotics, given the mild *Pasteurella* bacteremia without severe complications or neutropenia. Discharge was planned after completing intravenous therapy, with oral antibiotics as needed for otitis media. The oncologist determined that the next chemotherapy cycle could proceed after infection resolution, but delayed it by one week to allow completion of antibiotic therapy. Postdischarge, the patient underwent regular outpatient visits to monitor symptoms and hematologic status, with the otolaryngologist ensuring no progression of otitis media. After 10 days of intravenous antibiotic administration (ceftriaxone for three days and then ampicillin for seven days), the patient was discharged home with 250 mg of oral amoxicillin three times daily for an additional four days.

**Table 2:** Susceptibility test results of blood culture.

Antibiotics	Minimum inhibitory concentration	Susceptibility
Ampicillin	12	S
Penicillin G	12	S
Ceftriaxone	<12	S
Amoxicillin/Clavulanic	<25	S
Erythromycin	2	R
Azithromycin	0.5	S
Trimethoprim/Sulfamethoxazole	<0.5	S

## Discussion

This case report presented a rare combination of *P. multocida *infection characterized by otitis media with effusion and bacteremia in an active breast cancer patient without neutropenia. Our patient presented with fever, sore throat, and mild ear fullness. Given that the patient appeared stable except for fever, we considered the symptoms to be from viral upper respiratory infections that multiplex PCR could not detect. We performed blood cultures to evaluate for bacteremia, which does not always present with obvious signs of infection. The cultures later revealed *P. multocida*. Finally, the patient was diagnosed with otitis media with effusion and bacteremia, was prescribed antibiotics, and recovered without severe complications. This case indicates two key points. First, diagnosing *P. multocida* infection can be challenging when otitis media presents with only mild or nonspecific symptoms. Second, clinicians can diagnose and treat appropriately by integrating thorough animal contact history-taking, blood culture, and microbiological analysis.

*P. multocida *infections can vary widely in presentation, and mild symptoms may lead to initial misdiagnosis [1,3-7,11,12]. Ear complications associated with *P. multocida* are rarely reported. We conducted a literature search for *P. multocida*-associated otitis media using PubMed and Google Scholar databases up to January 18, 2024. The detailed search terms are provided in the Appendices. We identified 17 previously reported cases, resulting in a total of 18 cases (Table 3).

**Table 3:** Previous case reports on otitis media caused by Pasteurella infection. F: female; M: male; NA: not available; CSF: cerebrospinal fluid; (+): *P. multocida* growth from the culture; (-): no growth of *P. multocida* ^a^: Reported as *P. septica.* b: Ear swab culture revealed mixed growth of *Escherichia* *coli* and diphtheroids. ^c^: The dog pharynx culture revealed *P. multocida* of a different biotype than the patient’s. ^d^: Ear swab culture revealed the growth of *Klebsiella pneumoniae.*

Author(s) year	Age, sex	Underlying disease	Animal contact	Otitis media presentation, laterality	Complication	Blood culture	Other cultures	Treatment	Outcome, sequelae
Svendsen et al., 1947 [13]	18 M	NA	None	Acute exacerbations of chronic	Meningitis, brain abscess	NA	Brain abscess (+)^a^	Penicillin→penicillin, sulfadiazine	Survived, no sequelae
Holmes et al., 1965 [14]	34 F	NA	NA	Acute exacerbations of chronic	Pharyngitis, cervical lymphadenopathy	NA	NA	Penicillin, streptomycin, aerosporin	NA
Larsen et al., 1969 [15]	14 F	NA	NA	Acute exacerbations of chronic	Meningitis, brain abscess	NA	CSF (-), ear material (-)	Cloxacillin, penicillin, chloramphenicol	Survived, cerebellar symptoms remained
Easton et al., 1970 [16]	56 M	Pituitary adenoma	None	Acute, unilateral	Meningitis	NA	CSF (+), ear discharge (-)	Penicillin, chloramphenicol, sulfadimidine→ cloxacillin, chloramphenicol, sulfadimidine	Survived, no sequelae
McCue, 1979 [17]	16 F	None	Handling rabbit	Acute, left	Meningitis	(+)	CSF (+)	Penicillin, chloramphenicol→penicillin,	Survived, no sequela
Smith, 1980 [18]	35 M	None	Kept cats	Chronic	Meningitis	NA	CSF (+), ear swab (-)^b^	Ampicillin	Survived, no sequelae
Whittle and Besser, 1982 [19]	65 F	None	NA	Chronic, left	Cerebral abscess, glomus jugular tumor	NA	Brain abscess (+), ear discharge (+)	Penicillin, metronidazole, dexamethasone	Survived, no sequelae
Bruun and Friis-Moller, 1983 [20]	75 F	None	Kept dogs^c^	Acute exacerbations of chronic	Meningitis	(+)	CSF (+), ear swab (-) ^d^	Ampicillin, gentamicin→chloramphenicol→penicillin, cefotaxime	Survived, no sequelae
Fell, 1984 [21]	35 M	Remote skull fracture	Kept dogs, occupational exposure (slaughter)	Acute, left	Meningitis	NA	CSF (+)	Penicillin, chloramphenicol→penicillin	Survived, no sequelae
Rapp et al., 1990 [22]	50 M	Obesity and possible type II diabetes	Occupational exposure (farmer)	Acute, right	Bell’s palsy	(+)	Middle ear pus (+)	Ampicillin, trimethoprim, sulfamethoxazole	Survived, no sequelae
Li et al., 1994 [23]	26 M	None	Kept cats and dogs	Chronic	Brain abscess	NA	Brain abscess (+)	Ampicillin, chloramphenicol, gentamycin	Survived, no sequelae
Godey et al., 1999 [2]	67 M	Right middle-ear cholesteatoma, operated right mastoiditis	Licked by a dog	Acute exacerbations, chronic, right	Meningitis	NA	CSF (+), ear swab (+)	Cefotaxime, vancomycin→cefotaxime, amoxicillin, ofloxacin, mastoidectomy	Survived, no sequelae
Green et al., 2002 [11]	37 F	Multiple episodes of otitis media,	Licked by cats	Acute, bilateral	Meningitis	(+)	CSF (+) throat (-), sputum (-), ear (-)	Ceftriaxone, vancomycin→ ceftriaxone, gentamicin→ ceftriaxone	Survived, no sequelae
Tattevin et al., 2005 [24]	66 M	Alcoholism	Dog contact	Unknown details of otitis	Meningitis	NA	CSF (+), ear (+)	Cefotaxime, surgery	Survived, no sequelae
Jordan et al., 2007 [25]	60 F	Hypertension, sleep disorder	Cat bite	Acute, left	Meningitis	(+)	CSF (-), ear (+)	Azithromycin, ofloxacin (optic drops)→aztreonam, clindamycin→ aztreonam, levofloxacin→levofloxacin	Survived, slight hearing loss remained
Tjen et al., 2007 [26]	44 F	Temporary ileostomy for diverticular abscess	Licked by dogs	Acute	Meningo-encephalitis	(+)	NA	Ceftriaxone, acyclovir→benzylpenicillin	Survived, no sequelae
Larne et al., 2018 [27]	66 F	Immunocompetent	Feeding stray cats	Acute, right	Meningitis	(+)	NA	Ceftriaxone, ampicillin/sulbactam→rifampin, levofloxacin, trimethoprim, sulfamethoxazole	Survived, no sequelae
Our case, 2025	49 F	Breast cancer	Kept dogs	Acute, right	Cellulitis, lymphadenitis	(+)	None	Ceftriaxone→ ampicillin→amoxicillin	Survived, no sequelae

Among these 18 patients, nine had acute otitis media, three had chronic otitis media, and five had acute exacerbations of chronic otitis media. Almost half (8 out of 18 cases) did not have any prior history of ear disease. The scarce number of reports may reflect prior antibiotic use, minimal effusion that hinders definitive culture results [12], and subtle presentations that can lead to underdiagnosis. Despite its rarity, otitis media warrants attention in *P. multocida *infections because it can trigger severe sequelae if left untreated [11,12]. Overall, 16 (89%) of these 18 patients had central nervous system or cranial nerve involvement, including 13 with meningitis and four with brain abscess. Our patient noticed ear fullness following the onset of pharyngitis, performed an ear equalization maneuver, and then developed a mild headache. This presentation reflected an ascending infection from the throat to the middle ear, which is consistent with a previous report [22]. We did not perform a cerebrospinal fluid examination because of the mild symptoms and clinical improvement observed with treatment. At the time of writing, 10 months have passed since the onset of the disease, and the patient has not experienced deterioration or relapse. We believe that the early initiation of antibiotics during mild symptoms prevents the progression of meningitis. Our cases demonstrated the importance of caution for mild ear symptoms in cancer patients, especially when they have a history of animal exposure.

The prompt diagnosis of bacteremia is crucial but challenging, especially among cancer patients without neutropenia. The mortality of bacteremia reaches approximately 30% of patients with solid tumors [28]. Bacteremia often accompanies approximately half of *Pasteurella* infections in this population [6]. Our patient presented with no notable findings other than a sore throat and ear fullness, suggesting that pharyngitis was the source of bacteremia. However, diagnosing bacteremia was difficult during the initial emergency department assessment because the patient exhibited only mild symptoms, and although a CT scan was performed, no definitive infectious focus was identified. In our case, the clue for diagnosis was performing blood culture examinations and obtaining an animal contact history.

No well-defined blood culture criteria exist for cancer patients without neutropenia [9,10]. In patients with FN, clinicians decide to order blood cultures and empiric antibiotics based on risk assessment tools such as the Multinational Association of Supportive Care in Cancer (MASCC) and Clinical Index of Stable Febrile Neutropenia (CISNE) scores [8,29], but the validity of these scores remains unknown for non-FN patients. Only approximately 2.4% of cancer patients who visit the emergency department have FN [9]; thus, the vast majority present without neutropenia [9,10]. Although patients with malignancy had a high admission rate of 83%, a quarter of those with severe complications were discharged from the emergency department at the initial assessment [10]. This fact suggests that existing risk assessment methods remain inadequate for cancer patients without neutropenia.

Multiple prognostic factors in addition to hypotension have been implicated in serious complications in bacteremia. A prospective study in patients with solid tumors revealed that advanced disease and steroid use are associated with death [28]. The modified Shapiro criteria and the CEC SEPSIS KILLS pathway also include high fever, intravascular devices, suspected infective endocarditis, chills, vomiting, an abnormal respiratory rate, and abnormal blood gas results as sepsis risk indicators in addition to hypotension [30]. Notably, fever of unknown origin (FUO) accounts for 8% of visits by cancer patients to the emergency department [28]. Although FUO is not an established prognostic factor, it can suggest infective endocarditis under the modified Shapiro criteria or highlight an infection that triggers severe complications, as observed in this case. Our patient’s experience suggests that “unknown” or “unexplained” fever in a non-neutropenic patient can indicate potential bacteremia.

Furthermore, questioning immunocompromised patients about animal contact can prompt early diagnosis and treatment [1]. Many patients and their families do not consider licking or casual contact with animals to be relevant risk factors. As animal-related infections are rare, clinicians do not routinely ask about exposure [1,31]. However, case-control studies and molecular typing data strongly suggest animal origins for certain infections, which we may be underestimating [1]. Immunocompromised individuals, pregnant women, older adults, and neonates may acquire infections from saliva, fleas, lice, or contaminated environments, even without direct bites or scratches [1]. In addition to *Pasteurella* species, *Salmonella *and *Campylobacter* also pose zoonotic risks [1,32,33]. If these organisms cause isolated bacteremia, blood cultures provide the only evidence [7].

Therefore, clinicians should carefully monitor febrile cancer patients without neutropenia if they have intravascular devices, use steroids, have advanced disease, present with an unknown fever source, or have had animal contact. Bacteremia remains a possibility in these patients, even when their circulatory status appears stable.

## Conclusions

In immunocompromised hosts, *P. multocida *can cause diverse infections, including otitis media with effusion. When cancer patients present with unexplained fever, clinicians should consider asking about animal contacts and ordering blood cultures even if the patients do not have neutropenia or hypotension. Further research is needed to develop a risk assessment strategy for febrile cancer patients without neutropenia.

## Appendices

Literature search method

We conducted a literature search for P. multocida-associated otitis media using PubMed and Google Scholar databases up to January 18, 2024. Two reviewers (M.K. and K.I.) independently assessed the articles for inclusion. Duplicate articles and animal studies were excluded based on the title and/or abstract review. Case series with unclear details and non-English literature were excluded. We also examined case reports cited in the included articles. For PubMed, we used the search terms “Pasteurella multocida AND otitis media AND humans” or “Pasteurella otitis” with the “Humans” filter applied. For Google Scholar, we used the search term “in title: Pasteurella otitis.” We identified a total of 17 reported cases, along with our case, which are summarized in Table 3.
